# Methylenetetrahydrofolate Reductase (MTHFR) C677T and A1298C Polymorphisms in Georgian Females with Hypothyroidism

**DOI:** 10.1055/s-0040-1714091

**Published:** 2020-07-20

**Authors:** Tamar Kvaratskhelia, Elene Abzianidze, Ketevan Asatiani, Merab Kvintradze, Sandro Surmava, Eka Kvaratskhelia

**Affiliations:** 1 Department of Molecular and Medical Genetics, Tbilisi State Medical University, Tbilisi, Georgia; 2 Division of Endoctinology, Medical Center “Medimedi,” Tbilisi, Georgia; 3 Metabolic Disorders, National Institute of Endocrinology, Tbilisi, Georgia

**Keywords:** hypothyroidism, hyperhomocysteinemia, MTHFR, SNPs, PCR-RFLP

## Abstract

The aim of this study was to investigate the frequency of methylenetetrahydrofolate reductase ( *MTHFR)* gene polymorphisms in Georgian females with hypothyroidism. Thirty-four patients and 29 healthy individuals were recruited in this study. Polymerase chain reaction-restriction fragment length polymorphism analyses were used for genotyping of *MTHFR* polymorphisms. The results of this study suggest that the *MTHFR* C677T variant was significantly associated with hypothyroidism. In addition, in individuals with T allele risk of hypothyroidism significantly increased. Combination of CT/AA genotypes was more prevalent in the hypothyroid patients than in the control group. Thus, C677T polymorphism could be a possible genetic factor contributing to the pathophysiology of hypothyroidism, possibly through hyperhomocysteinemia.

## Introduction

Methylenetetrahydrofolate reductase (MTHFR) is the trifunctional enzyme catalyzing conversion of 5,10-methylenetetrahydrofolate to 5-methylenetetrahydrofolate, which in turn is the major circulatory form of the folate in the blood and the co-substrate for homocysteine (Hcy) remethylation to methionine. 1 The *MTHFR* gene is composed of 11 exons and located on the short arm of the first chromosome (1p36.3). 2 3 From a total of 65 polymorphisms, two common single nucleotide polymorphism (SNPs) of *MTHFR* , on the 677th and 1,298th positions, have been described as clinically relevant. 4 5 A common C677T polymorphism at exon 4 causes an amino acid substitution at position 222 from alanine to valine (A222V), thus making the enzyme thermolabile. In homozygous condition, its decreased activity causes the development of mild hyperhomocysteinemia. 6 7 Another common SNP is an A to C change in position 1298 (A1298C) at exon 7 causing a glutamate to alanine substitution on the 429th position, resulting in decreased MTHFR activity, which is not associated with thermolability of an enzyme. Decreased MTHFR activity is more pronounced in the homozygous state than the heterozygous, and it does not appear to cause hyperhomocysteinemia in either genotype, but its combination with the C677T polymorphism—compound heterozygosity—results in significant elevation of plasma Hcy, even more severe than in 677TT individuals. 8 9 10

On the other hand, several studies indicated a higher plasma Hcy levels in patients with hypothyroidism than in healthy, euthyroid individuals, including some experimental studies that indicated decreased MTHFR activity in hypothyroid patients. 11 Hypothyroidism is usually the primary hypofunction of the thyroid gland resulting in an increase of thyroid-stimulating hormone (TSH) levels in blood; it comprises of two types: subclinical hypothyroidism (ScH) and overt hypothyroidism (OH), according to the extent of decrease in thyroid function. 12 ScH is often asymptomatic, only 30% of the patients may reveal thyroid hormone deficiency symptoms. Also, ScH is identified in the presence of persistently raising TSH levels and normal free thyroxine (FT4). OH is defined by its clinical symptoms and high levels of TSH combined with decreased levels of thyroid hormone. 13 14 Hypothyroidism occurs more frequently in women than men. 15

We conducted this study to see whether there is an association between the *MTHFR* genes' two most common SNPs (C677T and A1298C) and nonautoimmune hypothyroidism.

## Materials and Methods

Ethical approval for the study was granted by the Ethics Committee of Tbilisi State Medical University. Informed written consent for the collection of blood and clinical data, as well as for genetic analysis was obtained from all participants. The study was proposed to subjects admitted to LTD medical center “Medimedi” and the National Institute of Endocrinology. The study group consisted of 34 female members. All patients were diagnosed based on serum levels of TSH, FT4, anti-TG (anti-thyroglobulin), and anti-TPO (antithyroperoxidase) antibodies. In all patients, TSH levels were elevated, with FT4 normal or decreased, and anti-TG and anti-TPO antibodies levels in normal reference ranges ( Table 1 ). For the control group, 29 healthy, female volunteers were recruited. None of the controls suffered from hypothyroidism nor had relatives with thyroid diseases.

**Table 1:** Clinical characteristics of the patients at the time of sampling

Variable	Study group	Control group
*n*	34	29
*Age*	52.7 ± 15.6	53.7 ± 15.2
*TSH mU/mL (N 0.3–4.5)*	11.1 ± 5	2.7 ± 1.1
*FT4 ng/d* L *(N 0.8–1.7)*	1.1 ± 0.3	1.2 ± 0.2
*Anti-TPO ab IU/mL (N < 34)*	8.5 ± 9	6.9 ± 7.2
*Anti-TG ab IU/mL (N < 100)*	23.7 ± 25.8	12.3 ± 15.4

Abbreviations: FT4, thyroxine; TG, thyroglobulin; TPO, thyroperoxidase; TSH, thyroid stimulating hormone.

Note: Values are presented as absolute numbers, mean ± SD.

2.5-mL blood samples were collected in ethylenediaminetetraacetic acid-coated blood collection tubes. Genomic deoxyribonucleic acid (DNA) was isolated from whole blood using DNA blood mini Kit (Qiagen) in accordance with the manufacturer's instructions. DNA was eluted in 200-µL elution buffer and stored at −20°C until further analysis.

Nucleic acid samples were checked for concentration by fluorometer (Qubit assays, Thermo Fisher Scientific, Waltham, Massachusetts, United States).

We used polymerase chain reaction (PCR)-restriction fragment length polymorphism analyses for genotyping *MTHFR* C677T and A1298C polymorphisms. For this, target sequences were amplified using PCR and the product was digested by the addition of restriction enzyme. Each 25-µL PCR reaction contained 100-ng genomic DNA, 0.2 μM of each primers and Hot Start Taq 2X Master Mix (New England BioLabs). The sequences of forward and reverse primers, PCR conditions, and restriction enzymes used are summarized in Table 2 .

**Table 2:** The primers, polymerase chain reaction conditions, and restriction enzymes used in this study

Gene	SNP	Primer pairs	PCR conditions	Restriction enzymes
*MTHFR*	C677T	5′-TGAAGGAGAAGGTGTCTGCGG-3′ 5′-AGGACGGTGCGGTGAGAGTG-3′	95°C for 5 min (95°C for 30 s, 60°C for 30 s, 72°C for 60 s) × 30 cycles 72°C for 10 min	HinfI
	A1298C	5′-CTTTGGGGAGCTGAAGGACTACTAC-3′ 5′-CACTTTGTGACCATTCCGGTTTG-3′	96°C for 5 min (96°C for 30 s 61°C for 30 s 72°C for 30 s × 40 cycles 72°C for 5 min	*Mbo* II

Abbreviations: PCR, polymerase chain reaction; SNP, single nucleotide polymorphism.

Digestion of PCR products with appropriate restriction enzymes was performed in 50 µL of total volume, using 25 μL of PCR product, 1-μL restriction enzyme, and 5-μL CutSmart buffer. The samples were incubated at 37°C for 15 minutes. The DNA samples were loaded in 3% agarose gel with addition of ethidium bromide (EtBr) that intercalates DNA and makes it visible under ultraviolet (UV) light. A DNA marker with fragments of known length and concentration was running through the gel at the same time as the samples. Electrophoresis was performed at 80 mV. Finally, the gel was photographed on an UV transilluminator (Enduro, Labnet Int., NJ, United States).

C677T polymorphism creates a recognition sequence for the HinfI restriction enzyme, it is detected by the digestion of a 198bp PCR product into two 23bp and 175bp fragments. The 23bp fragment is not visible on the agarose gel. Accordingly, CC wild genotype is characterized by the presence of a 198 bp fragment, TT homozygous polymorphic genotype by 175bp fragment, and CT heterozygous genotype by 198bp and 175bp fragments. There are two recognition sites for the *Mbo* II restriction enzyme in the A1298C polymorphism. After digestion, the most common AA genotype has three fragments: 28bp, 28bp, and 72bp. The CC homozygous polymorphic genotype is determined by the 100bp fragment and the AC heterozygous genotype by the presence of both 75bp and 100bp fragments.

Statistical analysis was performed using the Statistical Package for Social Sciences software version 23 (Chicago, Illinois, United States). The data are expressed as mean ± standard deviation (SD). The frequencies of the alleles and genotypes in patients and controls were compared using chi-square analysis. A *p* -value <0.05 was considered as statistically significant. We calculated odds ratio (OR) and 95% confidence intervals (CIs) in alleles: 677 C and T; 1298 A and C; Genotypes—677 CC, CT, TT, CT + TT; 1298 AA, AC, CC, AC + CC, and combined genotypes—677/1298 CC/AA, CC/AC, CC/CC, CT/AA, CT/AC, and TT/AA.

## Results

The distribution of C677T and A1298C polymorphism allelic, genotype and combined genotype frequencies in patients with hypothyroidism and healthy controls is shown in Table 3 .

**Table 3:** Distribution of *MTHFR* C677T and A1298C polymorphisms and allele frequencies

*MTHFR*	Genotypes and alleles	Study group *N* (%)	Controls *N* (%)	OR (95% CI)	*χ* ^2^ , *p-* Value
***C677T***		34	29		
	CC	16 (47.1)	25 (86.2)	0.14 (0.41–0.5)	10.55, 0.001
	CT	15 (44.1)	4 (13.8)	4.9 (1.4–17.3)	6.83, 0.009
	TT	3 (8.8)	0 (0)	2.8 (0.28–28.5)	0.82, 0.37
	CT + TT	18 (52.9)	4 (13.8)	7.03 (2.01–24.6)	10.55, 0.001
	C	47 (69.1)	54 (93.1)	0.17 (0.05–0.52)	11.32, 0.001
	T	21 (30.9)	4 (6.9)	6.03 (1.93–18.83)	11.32, 0.001
***A1298C***	AA	26 (76.5)	22 (75.9)	1.03 (0.32–3.31)	0.003, 0.955
	AC	6 (17.6)	4 (13.8)	1.34 (0.34–5.29)	0.174, 0.677
	CC	2 (5.9)	3 (10.3)	0.54 (0.08–3.49)	0.427, 0.514
	AC + CC	8 (23.5)	7 (24.1)	0.97 (0.30–3.09)	0.003, 0.955
	A	58 (85.3)	48 (82.8)	1.21 (0.46–3.14)	1.151, 0.698
	C	10 (14.7)	10 (17.2)	0.83 (0.32–2.15)	0.151, 0.698
***Combined genotypes***	CC/AA	11 (32.4)	20 (69)	0.22 (0.07–0.63)	8.394, 0.004
	CC/AC	3 (8.8)	2 (6.9)	1.31 (0.20–8.41)	0.080, 0.778
	CC/CC	2 (5.9)	3 (10.3)	0.54 (0.08–3.49)	0.427, 0.514
	CT/AA	12 (35.3)	2 (6.9)	7.37 (1.49–36.45)	7.302, 0.007
	CT/AC	3 (8.8)	2 (6.9)	1.31 (0.20–8.41)	0.080, 0.778
	CT/CC	0	0	–	–
	TT/AA	3 (8.8)	0	–	–
	TT/AC	0	0	–	–
	TT/CC	0	0	–	–

Abbreviations: CI, confidence interval; SD, standard deviation; OR, odds ratio.

Note: Values are presented as mean ± SD or %.

The frequencies of genotypes *MTHFR* 677CC, 677CT, and 677TT in patients and controls were 47.1, 44.1, 8.8%, and 86.2, 13.8, 0%, respectively. As for polymorphism A1298C, the patients and controls presented genotypes 1298AA, 1298AC, and 1298CC at the following frequencies: 76.5, 17.6, 5.9% and 75.9, 13.8, 10.3%, respectively. Also, we evaluated all possible genotype combinations according to C677T and A1298C polymorphisms in *MTHFR* —677CC/1298AA, 677CC/1298AC, 677CC/1298CC, 677CT/1298AA, 677CT/1298AC, 677CT/1298CC, 677TT/1298AA, 677TT/1298AC, and 677TT/1298CC; the frequencies in the case and control groups, respectively, were 32.4, 8.8, 5.9, 35.3, 8.8, 0, 8.8, 0, 0% and 69, 6.9, 10.3, 6.9, 6.9, 0, 0, 0, and 0%.

The current study shows an association of *MTHFR* C677T polymorphism with hypothyroidism as T allele (OR −6.03, 95% CI −1.93 to 18.83, *p* < 0.001), CT genotype (OR −4.9, 95% CI −1.4 to 17.3, *p* < 0.009), and 677/1298 CT/AA combined genotypes (OR −7.37, 95% CI −1.49 to 36.45, *p* < 0.007) is more prevalent in hypothyroid patients than in the control group. On the other hand, no association was found between A1298C polymorphism and developing hypothyroidism in the case and control individuals.

## Discussion and Conclusion

In this study, we investigated the association between the MTHFR polymorphisms (C677T and A1298C) and hypothyroidism in Georgian population. According to the data of National Center of Disease Control and Public Health of Georgia, 16 the absolute number of subclinical iodine-deficiency hypothyroidism and other forms of hypothyroidism diagnoses in 2018 was 35,357 and its prevalence was 939.7 per 100,000. Hypothyroidism affects 4 to 20% of the adult population, influenced by factors such as age, gender, race, body mass index, and dietary iodine intake. 17 It should be underlined that all metabolically active cells in the body are targets for thyroid hormones; this is the main reason of the multispectral pattern of hypothyroid condition. 18 Although detailed pathogenesis of nonautoimmune hypothyroidism remains unclear, blood-based biomarkers such as dietary folate and Hcy might play a role in the development of this condition. Morris et al, has reported evidence supporting the correlation between OH and increased serum Hcy concentration. 19 The mechanism underlying this correlation remains unknown; however, as inflammation is associated with thyroid dysfunction, oxidative stress induced by the elevated Hcy may be a risk factor for this disease.

MTHFR is a key enzyme in Hcy and folate metabolism. It catalyzes the conversion of 5,10-methylenetetrahydrofolate to 5-methyltetrahydrofolate, the predominant circulating form of folate. *MTHFR* gene polymorphisms can reduce the concentration of folate in serum, and mildly increase plasma total Hcy. 20 In the previous study, we found that the C677T SNP in the *MTHFR* gene may be a genetic risk factor for ScH. 21 Besides, epigenetic modifications such as DNA methylation may be involved in the pathophysiology of ScH. 22

The results of this case–control study suggest that the *MTHFR* C677T variant, but not A1298C, was significantly associated with hypothyroidism in Georgian women. In addition, in individuals with T allele risk of hypothyroidism significantly increased (OR −6.03, 95% CI −1.93 to 18.83, *p* < 0.001). We observed that combination of CT/AA genotypes was more prevalent in hypothyroid patients than in the control group (OR −7.37, 95% CI −1.49 to 36.45, *p* < 0.007). Thus, C677T polymorphism could be a possible genetic factor contributing to the pathophysiology of hypothyroidism possibly through hyperhomocysteinemia.

In summary, early identification of hypothyroidism individuals with *MTHFR* C677T polymorphism would be very informative for personalized treatment of this condition. We believe that Hcy lowering intervention (with Vitamin B6, B12, and folate) of selected (especially asymptomatic) patients, would be effective for the long-term outcome in treatment of hypothyroidism.
